# BFL: a node and edge betweenness based fast layout algorithm for large scale networks

**DOI:** 10.1186/1471-2105-10-19

**Published:** 2009-01-15

**Authors:** Tatsunori B Hashimoto, Masao Nagasaki, Kaname Kojima, Satoru Miyano

**Affiliations:** 1 Harvard College, Adams House, Cambridge, Massachusetts, MA 02138, USA; 2 Human Genome Center, Institute of Medical Science, University of Tokyo, 4-6-1 Shirokanedai, Minato-ku, Tokyo 108-8639, Japan

## Abstract

**Background:**

Network visualization would serve as a useful first step for analysis. However, current graph layout algorithms for biological pathways are insensitive to biologically important information, e.g. subcellular localization, biological node and graph attributes, or/and not available for large scale networks, e.g. more than 10000 elements.

**Results:**

To overcome these problems, we propose the use of a biologically important graph metric, betweenness, a measure of network flow. This metric is highly correlated with many biological phenomena such as lethality and clusters. We devise a new fast parallel algorithm calculating betweenness to minimize the preprocessing cost. Using this metric, we also invent a node and edge betweenness based fast layout algorithm (BFL). BFL places the high-betweenness nodes to optimal positions and allows the low-betweenness nodes to reach suboptimal positions. Furthermore, BFL reduces the runtime by combining a sequential insertion algorim with betweenness. For a graph with *n *nodes, this approach reduces the expected runtime of the algorithm to *O*(*n*^2^) when considering edge crossings, and to *O*(*n *log *n*) when considering only density and edge lengths.

**Conclusion:**

Our BFL algorithm is compared against fast graph layout algorithms and approaches requiring intensive optimizations. For gene networks, we show that our algorithm is faster than all layout algorithms tested while providing readability on par with intensive optimization algorithms. We achieve a 1.4 second runtime for a graph with 4000 nodes and 12000 edges on a standard desktop computer.

## Background

Advances in biotechnology have made it possible to collect vast amounts of genetic data. Although extensive research has been done on numerical and statistical methods to infer the relationship among genes, which we call gene networks, methods for analyzing such data visualizing large gene networks has received less attention.

There exists significant former literature on general graph layout algorithms such as orthogonal drawing, planar embedding, force-directed layout [1]. Similarly, metabolic networks with relatively small numbers of nodes (<100) have received significant attention, with notable algorithms being proposed by Karp [2], and [3]. However, these algorithms are designed with fundamentally different goals than those for gene networks. Well known fast graph-theoretic algorithms such as Sugiyama [4], radial tree [5] are capable of drawing large graphs, but give degenerate results for large and dense graphs. On the other hand, force based algorithms such as spring embedder [6] are able to produce symmetric and aesthetic results, but become intractable in the case of large datasets, and fail to represent biological datasets. Former work in incorporating biologically information [7,8] applies simple positional constraints, but do not scale well to large networks. It has also been noted that such algorithms fail to produce compact layouts [9].

Optimizing algorithms rely on minimizing an underlying metric, and have been used to great success. Grid-layout [3,9,10] has been used in cellular circuits to incorporate complex constraints, while multidimensional scaling [11] along with planar subgraph extraction [12], which maps an artificial metric to Euclidean space, has been used to create fast algorithms incorporating biological attributes.

All of the above approaches have their drawbacks; they either fail to reflect biological relationships in the layout or fail to scale for large problems. This problem arises because utilizing biological facts is a computationally expensive operation [9,10] which most algorithms are not designed for. Grid layout [13] for example, requires satisfying biologically meaningful component placements.

This paper introduces a fast, biologically relevant layout algorithm using the concept called betweenness.

Betweenness is most commonly used as a way of analyzing social networks [14,15]. This metric was first proposed by Freeman *et al*. [14] as a way to characterize sparsely connected graphs. Betweenness centrality for certain types of flow is known as an indicator of traffic through a certain node or edge [14,16,17]. The index has previously been used in ranking websites and clustering in social networks [15].

Biologically, betweenness is useful when the digraph relationship correspond to information flow. In this case modules from betweenness represents informationally isolated modules while the high betweenness nodes and edges are hubs and links with high betweenenss.

BFL is of interest in large gene, and protein networks. Protein and gene networks allow for a straightforward attack with BFL, in that a straightforward weighted layout will produce biologically relevant results, as they represent interaction networks. Metabolic networks on the other hand should first be analyzed with modularity analysis [11]. This is because the genes of interest are often not those with the highest information content (which would be common ATP, NADH pathways) but rather those which function uniquely.

Specifically, given *σ*_*st *_defined as the number of shortest paths between nodes *s *and *t*, and *σ*_*st*_(*v*) defined as the number of shortest paths passing through *v *(and *s, t *≠ *v*), betweenness is defined as the sum of *σ*_*st*_(*v*)/*σ*_*st *_for all nodes *s *and *t *in the graph. In other words, betweenness is the sum of the probabilities of *v *being in the shortest path between any two nodes. This definition of node betweenness has been extended to edges by Newman *et al*. as a way to extract community structures [15]. Edge betweenness is similarly defined as above by taking the sum of *σ*_*st*_(*e*)/*σ*_*st*_, where *σ*_*st*_(*e*) is defined as the number of shortest paths between *s *and *t *passing through edge *e*.

Recently, betweenness has become of interest in bioinformatics because of its biological importance in gene and protein networks. Specifically, it has been shown that betweenness values correctly identify bridge proteins [18], protein modules [11,16,19], and essential proteins [20].

Although there are other measures which fulfill the above measures, such as random walk betweenness [19] and eigenvector centrality [19], these measures have a higher runtime complexity and produce similar values. In some isolated cases, such as an extremely dense graph, these measures may result in better layout, although we consider that in general the runtime trade-off is unnecessary.

These results imply first that clusters generated with Girvan-Newman's algorithm [15] using edge betweenness accurately represent clusters in protein function [11]. Second, high betweenness value nodes are biologically important to the function of the gene network. Finally, betweenness based layout correctly identifies bridges, which is valuable to graph layout techniques. We attach a standard biological dataset by Luo *et al*. [21] to show these properties.

The remainder is organized as follows. In the methods section, we first define betweenness and then we demonstrate an efficient parallel algorithm for calculating betweenness. We then present a new node and edge betweenness based fast layout algorithm (BFL) and the specific score methods. Lastly, we present the expected runtime of the layout methods. In the results and discussion section, we show the effects of graph size and confirm the effectiveness of our approach on runtime. We then compare the run-times and outputs of various networks with other layout algorithms, and also show that betweenness is crucial to our algorithm.

## Methods

### Definition of betweenness

We will use the same notation originally developed by Brandes [17] to describe node and edge betweenness calculations. First, let *G *= (*V, E*) be a connected directed graph. We define *σ*_*ab *_to be the number of shortest paths between nodes *a *and *b *in *G*. We then define *σ*_*ab*_(*n*) as the number of shortest paths between *a *and *b *which go through *n *∈ *V*. In this paper, for each edge *e *∈ *E*, we denote *e*_*p *_and *e*_*c *_to be the originating and destination nodes respectively.

The node betweenness for node *v *is defined as

(1) $$\text{NB}(v)=\sum_{v_{i}\in V}\sum_{v_{j}\in V\backslash \{v_{i}\}}\frac{\sigma_{v_{i}v_{j}}(v)}{\sigma_{v_{i}v_{j}}}$$

The edge betweenness for edge *e *is defined as

(2) $$\text{EB}(e)=\sum_{v_{i}\in V}\sum_{v_{j}\in V\backslash \{v_{i}\}}\frac{\sigma_{v_{i}v_{j}}(e)}{\sigma_{v_{i}v_{j}}}$$

In order to calculate these betweennesses, Brandes [17] proposed an efficient backwards algorithm which starts at the leaf nodes of a tree of shortest paths from a source in V (we call the tree TSP) and accumulates the betweenness values to the root node. The following two properties of TSP is used in [17]:

(i) If the graph is a TSP, following property is satisfied for *a, b *∈ *V *with *a *as an ascendant of *b*.

(3) *σ*_*ab*_(*n*) = *σ*_*an*_·*σ*_*nb*_.

(ii) Similarly for each edge *e *∈ *E*, we define the sigma operator *σ*_*ab*_(*e*) to be the number of paths from *a *to *b *which pass through edge *e*. In a TSP, we have

(4) $$\sigma_{ab}(e)=\sigma_{ae_{p}}\cdot \sigma_{e_{c}b}.$$

We propose a new forward algorithm where we start at the root node and propagate downwards. This allows us to parallelize the operations in a much more straightforward way compared to the backwards algorithm as in the next section.

### Parallelized betweenness calculation

Brandes [17] previously showed an implementation for calculating edge betweenness values. We show that the forward algorithm operates upon the same principle while allowing for parallelism.

Given a graph *G*, we start by running Dijkstra's algorithm on each node *v *and storing all shortest paths from node *v *to all other nodes. This gives us a TSP *T *comprises of shortest paths from *v*.

Our algorithm attempts to break down the betweenness calculation for shortest paths starting at each node *v *in a recurrent relation.

The node betweenness NB(*v*) for a node *v *∈ *V *consists of the internal sum and the external sum (see Equation 1). Given the TSP *T *containing the shortest paths from *v *∈ *V*, we can obtain the internal sum of the node betweenness, i.e. $\sum_{v_{j}\in V/\{v\}}\frac{\sigma_{v_{i}v_{j}}(v)}{\sigma_{v_{i}v_{j}}}$. For this node *v*, we can derive a recursive relation for $\sigma_{v_{i}v_{j}}(v)$ in terms of the number of paths through its destination *k *as,

$$\frac{\sigma_{v_{i}v_{j}}(v)}{\sigma_{v_{i}v_{j}}}=\sum_{k}\left(\frac{\sigma_{v_{i}v}\sigma_{vk}}{\sigma_{v_{i}k}}\cdot \frac{\sigma_{v_{i}v_{j}}(k)}{\sigma_{v_{i}v_{j}}}\right).$$

The first term can be seen as the additional contribution made from the new edge between *v *and *k*. The latter term can be seen as the contributions of all nodes downstream of *k*. A proof of the correctness of the backwards form of this equation is given in [17].

We then derive the recursive formula for edge betweenness in a similar manner. Substituting Equation 1 into Equation 2, we can rewrite the betweenness of an edge *e *as:

$$\text{EB}(e)=\sum_{v_{i}\in V}\sum_{v_{j}\in V\backslash \{v_{i}\}}\frac{\sigma_{v_{i}e_{p}}\sigma_{e_{c}v_{j}}}{\sigma_{v_{i}v_{j}}}.$$

If we let *v *in the previous derivation equal to *e*_*p *_and *k *equal to *e*_*c*_, we have that

$$\text{EB}(e)=\sum_{v_{i}\in V}\sum_{v_{j}\in V\backslash \{v_{i}\}}\frac{\sigma_{v_{i}v}\sigma_{e_{p}e_{c}}}{\sigma_{v_{i}e_{c}}}\cdot \frac{\sigma_{v_{i}v_{j}(e_{c})}}{\sigma_{v_{i}v_{j}}}.$$

Since $\sigma_{e_{p}e_{c}}$ = 1, we have that

$$\text{EB}(e)=\sum_{v_{i}\in V}\sum_{v_{j}\in V\backslash \{v_{i}\}}\frac{\sigma_{v_{i}v}}{\sigma_{v_{i}e_{c}}}\cdot \frac{\sigma_{v_{i}v_{j}(e_{c})}}{\sigma_{v_{i}v_{j}}}.$$

This equation implies that the operations involved in calculating node betweenness can be used for edge betweenness values.

This algorithm can be parallelized for each *v *∈ *V *since the operations from each TSP *T *are independent. Therefore, this is a more efficient algorithm than many of the current methods, which depend on calculating node betweenness before edge betweenness.

The method presented in [17] relied upon this approach, but started at *v*_*j *_rather than *v*_*i*_. This is an obstacle for parallelization since the values of some *v*_*j *_cannot be fixed unlike those for *v*_*i*_.

For large networks, betweenness values become extremely large for central nodes, while terminal nodes with no children have zero centrality. In order to make this metric more suited for layout, we take the log centrality for both edge and betweenness (we add value one to the original betweenness value in order to avoid log(0)).

### Edge and node betweenness based fast layout algorithm BFL

After calculating the betweenness scores, our *edge and node betweenness based fast layout algorithm *(BFL) is executed as in Tables 1 and 2.

**Table 1:** Variable legend and overall layout algorithm

		Global Variables
Type	Name	Detail

Priority Queue	*Q*	Queue of nodes with largest node betweenness first
Array	*N odeBC *[*v*]	Betweenness values for node *v*
2D Array	*EdgeBC *[*v*_1_] [*v*_2_]	Edge betweenness from *v*_1 _to *v*_2_
Set	*S*	Empty set (contains nodes already placed)
Map	*H*	Empty multi-value map (Key – node, Value – orphans with *v *as parent)
Tree	*T*	Tree contains a quadtree containing nodes in Table 4

		Constants

double	*C*_1_	controls how far from the parent nodes are initially placed
double	*threshold*	lower values force tighter convergence constraints
double	*C*_2_	controls how far nodes are moved each iteration
integer	*k*_*max*_	controls when to cut off simulated annealing loops
double	*K*_1_	controls how much density is used in score calculation
double	*K*_2_	controls how much edge lengths are used
double	*K*_3_	controls how important edge crossings are
int	*maxT reeSize*	sets how large each bucket can be in the quadtree

**Table 2:** Fast Edge and Node Betweenness Based Layout Algorithm

1: **procedure **LAYOUT
2: Deque *v *← *Q*
3: Set *v. coordinate *← (0, 0)
4: Push *v *→ *S*
5: **while ***Q *is not empty **do**
6: Deque *v *← *Q*
7: **if ***S *contains a *v' *connected to *v ***then**
8: PlaceNode(*v*,*v'*)
9: **else**
10: **for all **Neighbors *n *of *v ***do**
11: Put (*n, v*) → *H*
12: **end for**
13: **end if**
14: **end while**
15: **end procedure**

16: **procedure **PLACENODE(*v*, *v'*)
17: Set *v. coordinate *← Coordinate generated by a Gaussian centered at *v'. coordinate *with variance *c*_1_*N odeBC *[*v*]^2^
18: Anneal(*v*,*threshold*)
19: Push *v *→ *S*
20: Get *nodes *← values in *H *with key *v *▹ Get orphans which can now be placed
21: **for all ***node *in *nodes ***do**
22: PlaceNode(*node*,*v*)
23: **end for**
24: **end procedure**

Table above shows the type and usage of each global variable as well as parameters

As described in introduction section, for the BFL layout algorithm, we mainly care following two points; (i) the important elements (high betweenness nodes and edges) should be emphasized in the resulting layout, (ii) the layout algorithm should run in real-time for large scale gene networks (around 10000 elements).

A naïve implementation of betweenness would scale scores as part of an optimizing algorithm. Such a naïve method was initially investigated, but incorporating betweenness in a global optimization algorithm caused significant slowdowns (conflicting with (ii)). The global optimization incurred a large penalty because the scaling forced high-betweenness nodes with strict tolerances to be optimized in a sea of lower-betweenness nodes.

For the above reason, instead of using a global optimization approach in BFL, we created a local optimization procedure which took advantage of the properties of betweenness and minimized the loss of quality. BFL places one node at a time in order of descending betweenness instead of placing all nodes at once (see lines 5 to 14 in Table 2). In BFL, simulated annealing is used to place the inserted node by minimizing the score function (the detail of this function is defined in the next section).

The execution steps of BFL in Tables 1 and 2 is summarized as follows. Initially, BFL stores all nodes to the priority queue *Q*, in which each node is prioritized with the node betweenness value (highest value is at the head) and creates an empty set *S *that is used to store the already placed nodes. In the first step, dequeue the top node in *Q*, put the node to *S*, and set the position of the node to (0,0). In the main recursive loop (lines 5 to 14), dequeue the current head node *v *in *Q *and check whether or not *S *contains a neighbor of *v*. (i) If *S *does not contain a neighbor, all edges connected to *v *are inserted into a map *H *(lines 9–13) and is not added the *v *to *S*. (ii) If *S *contains a neighbor of *v *(lines 7–9), the *v *is inserted to *S *(line 19) and one node *v' *in *S *is with the highest edge betweenness is selected and placed the *v *at the initial coordinate randomly drawn from a Gaussian centered at *v *with variance *c*_1_·*NodeBC*[*v*]^2 ^(line 17). The initial coordinate is optimized by using the annealing method described in the next section (line 18). In (ii), all nodes connected to *v *in *H *are also placed in the same manner (line 21). We separate execution branches (i) and (ii) in order to resolve orphan nodes. Since insertion order depends only on betweenness, there are some nodes which are disconnected from the currently placed graph *S*. In this case we put this node on a dependency queue, and place the node as soon as the dependency is fulfilled.

This significantly speeds up BFL layout since only nodes directly connected to the newly inserted node is needed to calculate the score function in each insertion step, i.e. pairwise effects elsewhere on the graph do not need to be calculated at all.

BFL runtime is further shorten by using following two properties. In the beginning phase, few nodes are already placed and score calculation proceeds quickly. Later, on the other hand, the inserted nodes will have few edges (since the value of betweenness is low), leading to much looser score tolerances and fewer children to process (the effectiveness of those properties are confirmed in later section with simulation test).

### Simulated annealing with betweenness based score function

For the insertion step of each node in BFL, we use simulated annealing to optimize the location of a newly inserted node *v*_*i *_∈ *V *by optimizing the following score function (which is referred to as EnergeOf in Table 3)

**Table 3:** Score functions.

1: **procedure **ENERGYOF(*v*,*currentCoord*)
2: Add *energy *← *c*_3 _density(*v*,*currentCoord*)
3: Add *energy *← *c*_4 _edgeLength(*v*,*currentCoord*)
4: Add *energy *← *c*_5 _edgeCrosses(*v*,*currentCoord*)
5: Return *energy*
6: **end procedure**

7: **procedure **DENSITY(*v*,*currentCoord*)
8: **for all ***node *in Set *s ***do**
9: Add *density *← $\frac{Log(NodeBC[node]+1)}{Distance{\left(v,node\right)}^{2}}$
10: **if ***v *and *node *overlap **then **11: Return ∞
12: **end if**
13: **end for**
14: Return *density*
15: **end procedure**

16: **procedure **EDGE LENGTH(*v*,*currentCoord*)
17: **for all **Edges of *v *connected to a *destination *in S **do**
18: Add *length *← *EdgeBC *[*v*] [*destination*] * Distance(*v, destination*)^2^
19: **end for**
20: Return *length*
21: **end procedure**

22: **procedure **EDGECROSSES(*v*,*currentCoord*)
23: **for all **Edges in *v *connected to a *destination *in S **do**
24: Add *crosses *← *CountIntersections*(*Edges*) * EdgeBC [*v*] [*destination*] ▹ Intersections should be counted with an efficent Ray-Shooting algorithm
25: **end for**
26: Return *crosses*
27: **end procedure**

Shows scoring functions for each aesthetic parameter, weighted by betweenness scores.

$$\text{Score}(v_{i},E_{v_{i}},{V^{\prime }}_{i},{E^{\prime }}_{i})=k_{1}\cdot \text{NodeDensity}(v_{i},{V^{\prime }}_{i})+k_{2}\cdot \text{EdgeLength}(E_{v_{i}})+k_{3}\cdot \text{EdgeCross}(E_{v_{i}},{E^{\prime }}_{i}),$$

where $E_{v_{i}}$ is the connected edges to the node *v*_*i*_, ${G^{\prime }}_{i}=({V^{\prime }}_{i},{E^{\prime }}_{i})$ is the subgraph before inserting node *v*_*i*_, and *k*_1 _+ *k*_2 _+ *k*_3 _= 1 (NodeDensity, EdgeLength and EdgeCross are defined later).

Similar metrics have been used in [22] and there have been aesthetic justifications for their use. Simulated annealing is even more suited in this case because of its robustness and single-point performance. While there are very efficient algorithms such as genetic, particle swarm, or ant colony optimization for parallel optimization procedures, BFL reduces global optimization to a series of local optimization problems, which removes the need for parallel optimization. In this case, nearly all stochastic optimization problems become a variant of simulated annealing. On the other hand, hill climbing and BFGS based numerical optimization procedures are not robust enough for this problem. The optimization landscape is extremely multimodal (as each vertex becomes a local mode) and therefore the chance of local minima are extremely high.

In our score function, values of node and edge betweennesses are effectively used to ensure that high betweenness nodes are given more emphasis than low-betweenness ones with low calculation cost.

#### Node density function

In addition to the traditional notion that high node density makes graphs hard to read, we concluded that high betweenness nodes should contribute more to the local density score than low betweenness nodes. We therefore define a density function for placing *v*_*i *_into a set of already placed nodes ${V^{\prime }}_{i}$

$$\text{NodeDensity}(v_{i},{V^{\prime }}_{i})=\sum_{{v^{\prime }}_{i}\in {V^{\prime }}_{i}}\left||v_{i}-{v^{\prime }}_{i}||\cdot \text{NB}(v_{i})\cdot \text{NB}({v^{\prime }}_{i})(\text{correspond to Table }3,\text{ lines }7\text{-}15\right),$$

where $\text{NB}(v)=\sum_{v_{i}\in V}\sum_{v_{j}\in V\backslash \{v_{i}\}}\frac{\sigma_{v_{i}v_{j}}(v)}{\sigma_{v_{i}v_{j}}}$ is the Euclidean distance of nodes *v*_*i *_and ${v^{\prime }}_{i}$.

The density function will create a multi-scale layout; high betweenness nodes are separately positioned as core nodes and low-betweenness nodes are positioned around them.

We can efficiently implement a localized variant of this by using quad-trees (see Table 4). For a graph with *i *nodes, we can query a bucket in log(*i*) amortized runtime. [23]

**Table 4:** Fast density modifications

1: **procedure **FASTDENSITY(*v*,*currentCoord*)
2: Set *currentNode *← head of tree *T*
3: **while ***currentNode *is not a leaf **do**
4: **if ***currentCoord > currentNode.vpartition ***then**
5: **if ***currentCoord > currentNode.hpartition ***then**
6: Set *currentNode ← currentNode.topRight*
7: **else **Set *currentNode ← currentNode.bottomRight*
8: **end if**
9: **else**
10: **if ***currentCoord > currentNode.hpartition ***then**
11: Set *currentNode ← currentNode.topLeft*
12: **else **Set *currentNode ← currentNode.bottomLeft*
13: **end if**
14: **end if**
15: **end while**
16: **for all ***node *in Set *currentNode ***do**
17: Add *density *← $\frac{Log(NodeBC[node]+1)}{Distance{\left(v,node\right)}^{2}}$
18: **if ***v *and *node *overlap **then**
19: Return ∞
20: **end if**
21: **end for**
22: Return *density*
23: **end procedure**
24: **procedure **FASTPLACENODE(*v*,*v*^'^)
25: Set *v.coordinate *← Coordinate generated by a Gaussian centered at *v'.coordinate *with variance *c*_1_*N odeBC *[*v*]^2^
26: Anneal(*v*,*threshold*)
27: Push *v → S*
28: InsertNode(v) ▹ Node insertion to tree added
29: Get set of *nodes ← *values in *H *with key *v *▹ Get orphans which can now be placed
30: **for all ***node *in *nodes ***do**
31: PlaceNode(*node*,*v*)
32: **end for**
33: **end procedure**
34: **procedure **INSERTNODE(*v*)
35: Set *currentN ode ← *head of tree *T*
36: **while ***currentN ode *is not a leaf **do**
37: **if ***currentCoord > currentN ode.vpartition ***then**
38: **if ***currentCoord > currentN ode.hpartition ***then**
39: Set *currentN ode ← currentN ode.topRight*
40: **else **Set *currentN ode ← currentN ode.bottomRight*
41: **end if**
42: **else**
43: **if ***currentCoord > currentN ode.hpartition ***then**
44: Set *currentN ode ← currentN ode.topLef t*
45: **else **Set *currentN ode ← currentN ode.bottomLef t*
46: **end if**
47: **end if**
48: **end while**
49: *currentN ode.add*(*v*)
50: **while ***currentN ode.size > maxT reeSize ***do**^22^
51: Set *currentN ode.topRight ← currentN ode.partitionT opRight*
52: Set *currentN ode.topLeft ← currentN ode.partitionT opLef t*
53: Set *currentN ode.bottomRight ← currentN ode.partitionBottomRight*
54: Set *currentN ode.bottomLeft ← currentN ode.partitionBottomLef t*
55: **end while**
56: **end procedure**

This pseudo code shows modifications needed to query local densities and speed overall runtime.

#### Edge length function

In [22], the average edge length is used to counterbalance the density and prevent a space-inefficient layout. In BFL, each edge length is scaled by its betweenness score, which forces nodes to shorten high betweenness edges over low betweenness ones. Edge lengths therefore as a aesthetic measure of the contribution of an edge to the node betweenness.

We define an edge length function for edges $E_{v_{i}}$ connected to the newly inserted node *v*_*i*_,

$$\text{EdgeLength}(E_{v_{i}})=\sum_{e_{v_{i}}\in E_{v_{i}}}\left||e_{v_{i}}||\cdot \text{EB}(e_{v_{i}})(\text{correspond to Table }3,\text{ lines }16\text{-}21\right),$$

where $\left||e_{v_{i}}|\right|$ is the Euclidean length of the edge $e_{v_{i}}$.

#### Edge crossing function

In order to achieve (i), the important elements should be emphasized in the resulting layout. Edge crossings high betweenness edges should be minimized, while crossings among low betweenness edges can be tolerated (for the sake of runtime). For this reason, each edge crossing is scaled by its betweenness score. Similarly, for newly inserted edges $E_{v_{i}}$, we define an edge crossing function,

$$\text{EdgeCross}(E_{v_{i}},{E^{\prime }}_{i})=\sum_{e_{v_{i}}\in E_{v_{i}}}\sum_{{e^{\prime }}_{i}\in {E^{\prime }}_{i}\backslash \{e_{v_{i}}\}}\delta (e_{v_{i}},{e^{\prime }}_{i})\cdot \text{EB}({e^{\prime }}_{i}\text{)}\cdot \text{EB}(e_{v_{i}})\;(\text{correspond to Table }3,\text{ lines }22\text{-}24),$$

where $\delta (e_{v_{i}},{e^{\prime }}_{i})$ is an indicator function which returns 1 if $\text{EdgeCross}(E_{v_{i}},{E^{\prime }}_{i})=\sum_{e_{v_{i}}\in E_{v_{i}}}\sum_{{e^{\prime }}_{i}\in {E^{\prime }}_{i}\backslash \{e_{v_{i}}\}}\delta (e_{v_{i}},{e^{\prime }}_{i})\cdot \text{EB}({e^{\prime }}_{i}\text{)}\cdot \text{EB}(e_{v_{i}})\;(\text{correspond to Table }3,\text{ lines }22\text{-}24),$ and ${e^{\prime }}_{i}$ cross and otherwise returns 0. In order to calculate the number of crossings, we use the efficient ray shooting algorithm proposed by [24].

For our simulated annealing loop, a polynomial cooling scheme is specified by defining the temperature *t *as

*t *= (*k*_*max *_- *k*)^*n*^,

where *k*_*max *_is the maximum iteration count and *k *is the current loop (correspond to Table 5, lines 24–33). Former literature [22] and our tests suggested that *n *= 3 was reasonable for most cases.

**Table 5:** Annealing and optimization algorithm

1: **procedure **ANNEAL(*v*,*threshold*)
2: Set *currentCoord ← v.coordinate*
3: Set *currentEnergy *← EnergyOf(*v, currentCoord*)
4: Set *bestCoord ← currentCoord*
5: Set *bestEnergy ← currentEnergy*
6: Set *k *← 0
7: **while ***currentEnergy – tempEnergy > threshold *AND *k < kmax ***do **▹ *kmax *is some constant to cut off runaway calculations
8: Set *tempCoord *← newNeighbor(*currentCoord*,*v*)
9: Set *tempEnergy *← EnergyOf(*v*,*tempCoord*)
10: **if ***bestEnergy > tempEnergy ***then**
11: Set *bestEnergy ← tempEnergy*
12: Set *bestCoord ← tempCoord*
13: **end if**
14: **if **transition(*currentEnergy, tempEnergy, k*) **then**
15: Set *currentCoord ← tempCoord*
16: Set *currentEnergy ← tempEnergy*
17: **end if**
18: **end while**
19: Set *v.coordinate ← bestCoord*
20: **end procedure**

21: **procedure **NEWNEIGHBOR(*currentCoord*,*v*)
22: Return Gaussian centered at *currentCoord *with deviation *c*_2_*Ln*(*N odeBC *[*v*] + 1)^3^
23: **end procedure**

24: **procedure **TRANSITION(*currentEnergy*,*tempEnergy*,*k*)
25: Set *temp ← *(*kmax *- *k*)^3^
26: Set *transition ← e*^(*currentEnergy*-*tempEnergy*)/*temp*^
27: Set *rand *← random value from 0 to 1
28: **if ***rand < transition ***then**
29: Return true
30: **else**
31: Return false
32: **end if**
33: **end procedure**

General annealing implementation is shown here with a cubic cooling schedule.

### Layout algorithm runtime analysis

Since the betweenness calculation can be cached into the network file for repeated uses, we only consider the runtime of the layout algorithm itself. The runtime of the layout is dominated by evaluation of the scoring function which is called |*V*| times.

Let *f *be the runtime of scoring function. The total runtime is given as

$$\sum_{i=0}^{|V|-1}f(v_{i},E_{v_{i}},{V^{\prime }}_{i},{E^{\prime }}_{i}).$$

While nodes are sequentially inserted according to the value of the node betweenness, we cannot know the exact values of |$E_{v_{i}}$|, |${V^{\prime }}_{i}$|, and |${E^{\prime }}_{i}$| in advance. Thus, we evaluate the expected total runtime to analyze its asymptotic behavior,

(5) $$E\left(\sum_{i=0}^{|V|-1}f(v_{i},E_{v_{i}},{V^{\prime }}_{i},{E^{\prime }}_{i})\right)=\sum_{i=0}^{|V|-1}E(f(v_{i},E_{v_{i}},{V^{\prime }}_{i},{E^{\prime }}_{i})).$$

The runtime of the score function can be expressed by the sum of its component run-times, which is

$$f(v_{i},E_{v_{i}},{V^{\prime }}_{i},{E^{\prime }}_{i})=\text{NodeDensity}(v_{i},{V^{\prime }}_{i})+\text{EdgeLength}(E_{v_{i}})+\text{EdgeCross}(E_{v_{i}},{E^{\prime }}_{i}).$$

The runtime for the first term NodeDensity takes *O*(log(|${V^{\prime }}_{i}$|)) since the quad-tree based density calculation method takes *O*(log(|${V^{\prime }}_{i}$|)) time to query the bucket and sum all the nodes [23]. The second term EdgeLength takes *O*(|$E_{v_{i}}$|) time to query all new edges. The last term EdgeCross is a ray-shooting problem which can be solved in $O(\sqrt{\left|{E^{\prime }}_{i}\right|}\log{\left(|{E^{\prime }}_{i}|\right)}^{2})$ time [24]. Thus, the expected total runtime in Equation 5 can be given as:

$$\begin{array}{c} \sum_{i=0}^{|V|-1}E(f(v_{i},E_{v_{i}},{V^{\prime }}_{i},{E^{\prime }}_{i}))=\sum_{i=0}^{|V|-1}E(\text{NodeDensity}(v_{i},{V^{\prime }}_{i})+\text{EdgeLength}(E_{v_{i}})+\text{EdgeCross}(E_{v_{i}},{E^{\prime }}_{i})).\\ =\sum_{i=0}^{|V|-1}\left(E(\log(|{V^{\prime }}_{i}|)+E(|E_{v_{i}}|)+E(\sqrt{\left|{E^{\prime }}_{i}\right|}\log{\left(|{E^{\prime }}_{i}|\right)}^{2})\right). \end{array}$$

$$=\sum_{i=0}^{|V|-1}\left(\log(i)+E(|E_{v_{i}}|)+E(\sqrt{\left|{E^{\prime }}_{i}\right|}\log{\left(|{E^{\prime }}_{i}|\right)}^{2})\right).$$

Since **E**(log(|${V^{\prime }}_{i}$|) is log(*i*), we remove the expectation of the first term to get,

$$=\sum_{i=0}^{|V|-1}\left(\log(i)+E(|E_{v_{i}}|)+E(\sqrt{\left|{E^{\prime }}_{i}\right|}\log{\left(|{E^{\prime }}_{i}|\right)}^{2})\right).$$

The expectation of |$E_{v_{i}}$| must be the average degree *d *of graph *G *since **E**(|$E_{v_{i}}$|) is the expected number of edges, which the newly inserted node *v*_*i *_brings. This leaves the expectation of $\sqrt{\left|{E^{\prime }}_{i}\right|}$. Since $\sqrt{\left|{E^{\prime }}_{i}\right|}$ is concave, by Jensen's inequality and **E**(|${E^{\prime }}_{i}$|) = *di *we have,

$$E(\sqrt{\left|{E^{\prime }}_{i}\right|}\log{\left(|{E^{\prime }}_{i}|\right)}^{2})<\sqrt{E(|{E^{\prime }}_{i}|)}\log{\left(E(|{E^{\prime }}_{i}|)\right)}^{2}=\sqrt{di}\log{\left(di\right)}^{2}.$$

Which dominates the density term, giving us an asymptotic runtime of

$$\sum_{i=0}^{|V|-1}\left(d+\sqrt{di}\log{\left(di\right)}^{2}\right)=d|V|+\sum_{i=0}^{|V|-1}\sqrt{di}/\log{\left(di\right)}^{2}.$$

We claim that the expression

$$\sum_{i=0}^{n}\sqrt{i}\log{\left(i\right)}^{2}=\zeta^{\left(2,0\right)}(-1/2,0)-\zeta^{\left(2,0\right)}(-1/2,n+1),$$

where *ζ*^(*x*,*y*) ^is the 2nd derivative of generalized Zeta function with respect to *x*.

*Proof of claim*. The generalized Zeta function is given by

$$\zeta (x,y)=\sum_{i=0}^{\infty }\frac{1}{{\left(i+y\right)}^{x}}$$

Taking the second derivative with respect to *x*,

$$\zeta^{\left(2,0\right)}(x,y)=\sum_{i=}^{\infty }{\left(i+y\right)}^{-x}\log{\left(i+y\right)}^{2}.$$

Plugging *x *= -1/2,

$$\zeta^{\left(2,0\right)}(-1/2,y)=\sum_{i=0}^{\infty }\sqrt{i+y}\log{\left(i+y\right)}^{2}.$$

$$\begin{array}{c} \zeta^{\left(2,0\right)}(-1/2,0)-\zeta^{\left(2,0\right)}(-1/2,n+1)=\sum_{i=0}^{\infty }\sqrt{i}\log{\left(i\right)}^{2}-\sum_{i=0}^{\infty }\sqrt{i+n+1}\log{\left(i+n+1\right)}^{2}\\ =\sum_{i=0}^{n}\sqrt{i}\log{\left(i\right)}^{2} \end{array}$$

Now by simple manipulation we can take into account the degree and make   □

$$\begin{array}{c} \sum_{i=0}^{|V|-1}\sqrt{di}\log{\left(di\right)}^{2}=\sqrt{d}\left(\sum_{i=0}^{|V|-1}{\left(\log(d)+\log(i)\right)}^{2}\right)\\ =\sqrt{d}\left(\sum_{i=0}^{|V|-1}\sqrt{i}\log{\left(d\right)}^{2}+\sum_{i=0}^{|V|-1}\sqrt{i}\log{\left(i\right)}^{2}+2\sum_{i=0}^{|V|-1}\log(d)\log(i)\right)\\ =\sqrt{d}\left(\log{\left(d\right)}^{2}\sum_{i=0}^{|V|-1}\sqrt{i}-2\log(d)(\zeta^{\left(1,0\right)}(-1/2,0)-\zeta^{\left(1,0\right)}(-1/2,|V|))-\zeta^{\left(2,0\right)}(-1/2,0)-\zeta^{\left(2,0\right)}(-1/2,|V|)\right). \end{array}$$

Removing negligible terms, we have

$$≃-\sqrt{d}\zeta^{\left(2,0\right)}(-1/2,|V|)+\sqrt{d}\log{\left(d\right)}^{2}\sum_{i=0}^{|V|-1}\sqrt{i}.$$

Using L'hopitel's rule and the knowledge that *ζ*^(2,1)^(-1/2, |*V*|) is zero,

$$\underset{|V|\to \infty }{\lim}\frac{-\zeta^{\left(2,0\right)}(-1/2,|V|)}{|V{|}^{2}}=\underset{|V|\to \infty }{\lim}\frac{-\zeta^{\left(2,1\right)}(-1/2,|V|)}{2|V|}=0.$$

Which shows that, the runtime grows slower than *d *log(*d*) with respect to degree *d *and slower than |*V*|^2 ^for node size |*V*|, or in little o notation,

*ζ*^(2,0)^(-1/2, |*V*|) = *O*(|*V*|^2^).

The estimated result *O*(|*V*|^2^) implies that our algorithm has an asymptotic complexity better than many fast optimizing algorithms with respect to node size. Edge crossing calculation can be ignored in many cases leading to an even faster runtime log(Gamma(|*V*| + 1)) if degree is constant, which is asymptotically equal to |*V*|log(|*V*|) and is the current standard for the fastest layout algorithms.

This speedup would not be possible without the sequential layout from the betweenness algorithm.

## Results and discussion

### Methods and datasets

The algorithm was implemented in Java with files stored in Cell System Markup Language (CSML) format [25]. A Fibonacci heap was used for the priority queue, all other data structures used library implementations available in the JDK.

Runtime tests were done on a 8-core Intel Xeon 4800 X5450 3 GHz machine with 16 GBs RAM, with random graphs generated by methods given by Rodionov *et al*. [26]. Comparisons to other programs were made on one sparse graph (2000 nodes and 7000 edges), two dense graphs (2000 nodes and 11000/47000 edges) and one estimated gene regulatory network (1897 nodes and 2849 edges) on an Athlon X2 3.3 GHz machine with 4 GBs RAM running on Windows XP. For the last gene regulatory network (calls UO Analysis), the microarray data of the ultradian oscillation (UO) clock in mouse presomitic mesoderm cells by Dequeant *et al *[27] is used. We generated graphs in CSML, GML, NET, and TLP files for various programs and used the same graph to compare run-times and outputs. These graph data files used in our simulation are available in the additional file 1.

For all tests, cached costs were not calculated (including loading, preprocessing, and betweenness calculations), as we are concerned with the time to layout.

### BFL runtime dependency on node size

**Figure 1:**
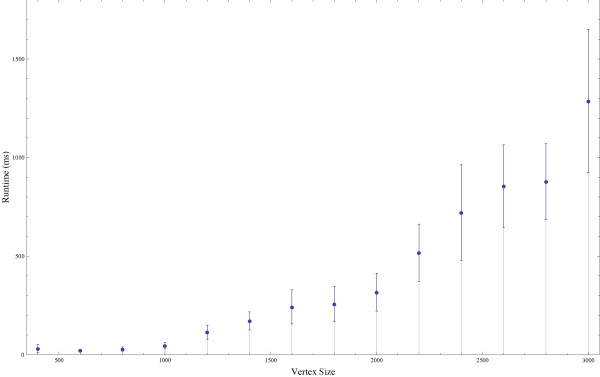
**Runtime changes from graph size**. Result of running the BFL algorithm on graphs of degree 3 ranging from 400 to 3000 nodes and measuring runtime.

Runtime for random graphs of degree three from size 400 to 3000 are shown in Figure 1. As the cores could not be load-isolated, the runtime fluctuations at the larger sizes are a result of parallel loads on the cluster. Even with the fluctuations however, the maximum runtime for layout is less than 13 seconds for a graph with 3000 nodes and 9000 edges. Betweenness calculations take more time, but such calculations were cached for this test since we evaluate solely the performance of the layout algorithm. The runtime graph seems to show that for constant degree, runtime rises near linearly from 1000 nodes to 3000 nodes, which is consistent with our previous analysis that the runtime for layout should be less than *n*^2^. We also note that the error bars grow, which is to be expected as the larger graphs have high variability with respect to graph structure and therefore can have highly unbalanced graphs, leading to longer run-times.

### BFL runtime dependency on betweenness

**Figure 2:**
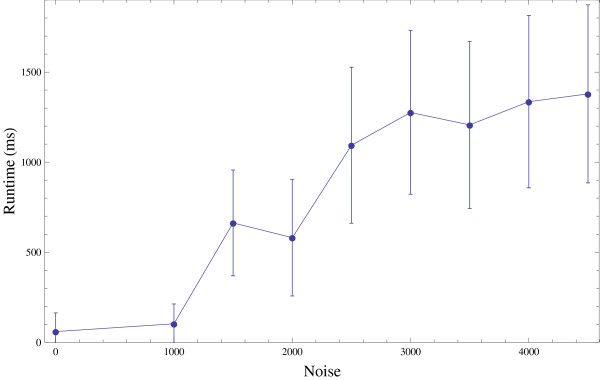
**Noise perturbation to betweenness**. Run-times of the algorithm when the betweenness information is perturbed. Runtime grows with noise.

In order to show that sequential insertion with betweenness order leads to a lower runtime, we corrupted the betweenness values with zero-mean fixed-variance Gaussian noise prior to ordering the nodes in the queue. Figure 2 shows the effect of such perturbation on runtime for a graph of 2000 nodes and 4000 edges. The runtime shows expected behavior as the runtime jumps when Gaussian noise becomes large enough to cause perturbations in the large-betweenness nodes. We also note that 1000-variance noise (which is relatively small, as the log betweenness is about seven) caused zero losses in runtime, and therefore future algorithms could use a heuristic version of betweenness calculated by random walk or approximative methods. Our algorithm would perform as well given low-noise approximations.

### BFL runtime dependency on graph density

While BFL is optimized for sparse networks, we show that BFL performance may actually improve with dense networks. In is not usual that the most of the nodes of the biological networks have high degrees. Instead, this is reasonable to consider parts nodes have high degrees in the network since they work as hub genes in the network [28]. Thus, we have applied our layout algorithms to the following graph. 10% of the node has high degree *m *(*m *is around 10% to the total node *n*). For a random graph with 2000 nodes (= *n*) and 7000 edges, we have created two graphs by adding (i) 4000 edges to 10% nodes (i.e. 200 nodes around degree 20 (= *m*)) and (ii) 40000 edges to 10% nodes (i.e. 200 nodes around degree 200 (= *m*)). For those graphs as in Table 6, the runtimes are reasonable as (i) 0.05 s and (ii) 3.7 s.

**Table 6:** Runtime Comparisons

	Runtime Comparisons Between Algorithms				
Dataset Number of Node/Edge	Random Graph 2000/7000	Random Graph 4000/12000	UO Analysis 1897/2849	Dense Graph 2000/11000	Dense Graph 2000/47000

Betweenness	.4s	1.4s	.8s	.05s	3.7s
Kamada-Kawai (Pajek)	17.9s	40.3s	22.2s	14.16s	32.64s
Fruchterman Reingold (Pajek)	30s	34s	31s	33.20s	42.06s
GEM (Tulip)	485s	1800s+	665s	394.95s	475s
RSFDP (InterViewer)	1.8s	2.1s	2.1 s	31.22s	40.46s

Runtime of the proposed method is compared to those fo existing methods (Kamada-Kawai, Fruchterman Reingold, GEM, and RSDFP) for several graphs. Dense Graph 2000/11000 and Dense Graph 2000/47000 were generated by adding 4000 and 40000 edges respectively to 10% nodes in Random Graph 2000/7000.

### BFL compared to existing algorithms

Table 6 shows the various run-times of our algorithm against those of four other competing algorithms. Force-directed and optimization algorithms similar to our own were chosen from possible candidates. By this criterion we compared against Kamada-Kawai [29] and Fruchterman Reingold [30] energy based algorithms in Pajek [31], GEM (Generalized Expectation-Maximization) based optimization in Tulip [32], and the RSDFP layout algorithm in InterViewer [33]. It is worth noting that our algorithm is implemented in Java while the competing algorithms are native applications. Thus, if our application were implemented efficiently in C, we would be able to achieve even faster run-times with even more drastic results. A future goal is to implement K-K, FR or GEM algorithms using the sequential insertion and betweenness weight functions used in the BFL algorithm. We hope to be able to get true force-directed algorithms which can produce better results with no increase in runtime.

### Resulting Layout of BFL compared to those of others

**Figure 3:**
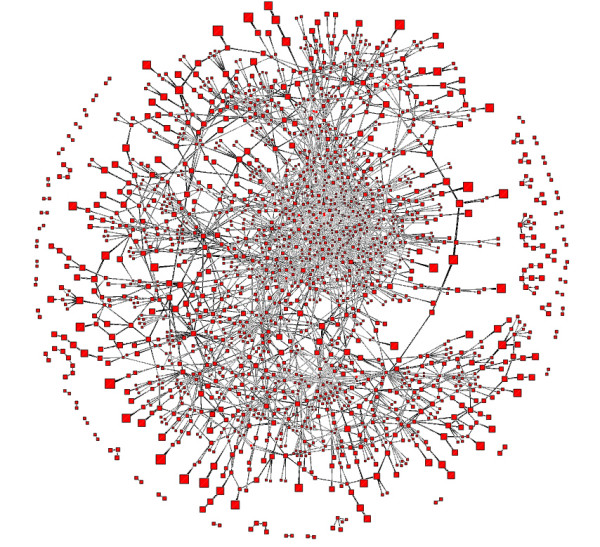
**Reference Layout: Tulip Running GEM**. A layout result of UO analysis network run on Tulip using the GEM algorithm.

**Figure 4:**
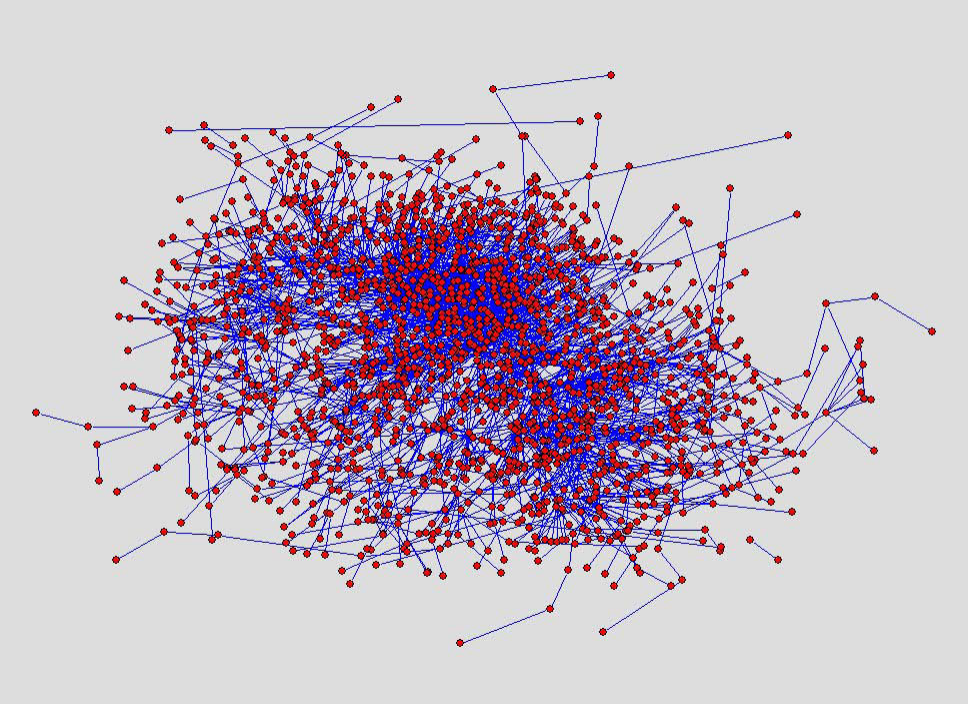
**Reference Layout: Pajek Running Kamada-Kawai**. A layout result of UO analysis network run on Pajek using Kamada-Kawai energy based algorithm.

**Figure 5:**
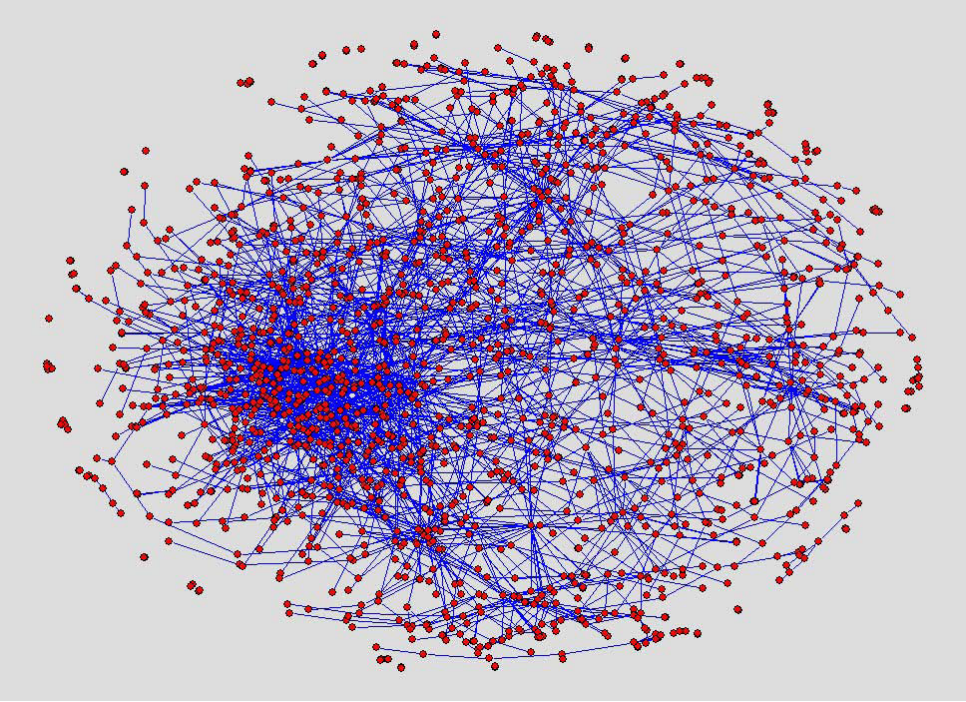
**Reference Layout: Pajek Running Fruchterman Reingold**. A layout result of UO analysis network run on Pajek using Fruchtermon Reingold energy based algorithm.

**Figure 6:**
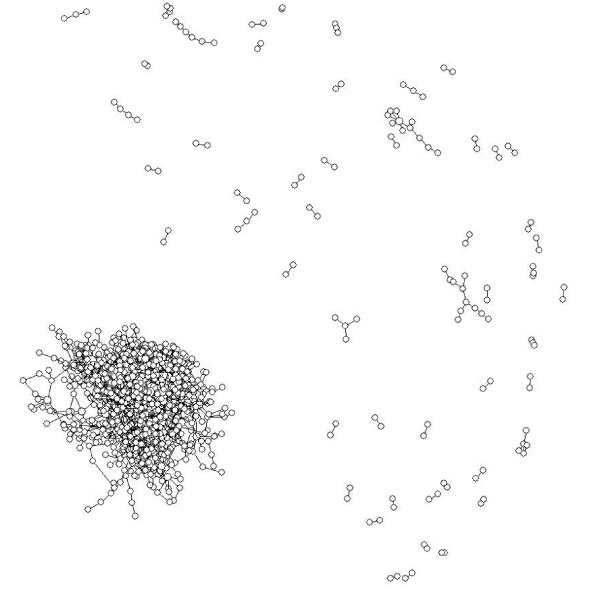
**Reference Layout: InterViewer Running RSFDP**. A layout result of UO analysis network run on InterViewer using the RSFDP algorithm.

**Figure 7:**
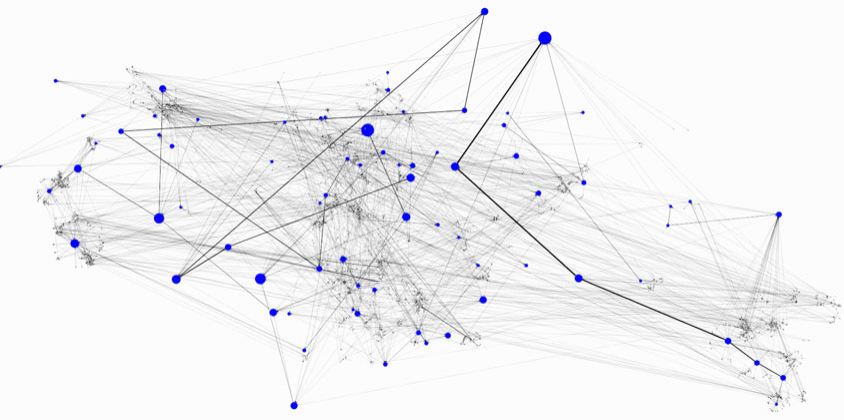
**Layout Result:Betweenness algorithm**. Result of the Betweenness based algorithm run on the UO analysis network red cutout refers to the enlarged section in Figure 8.

**Figure 8:**
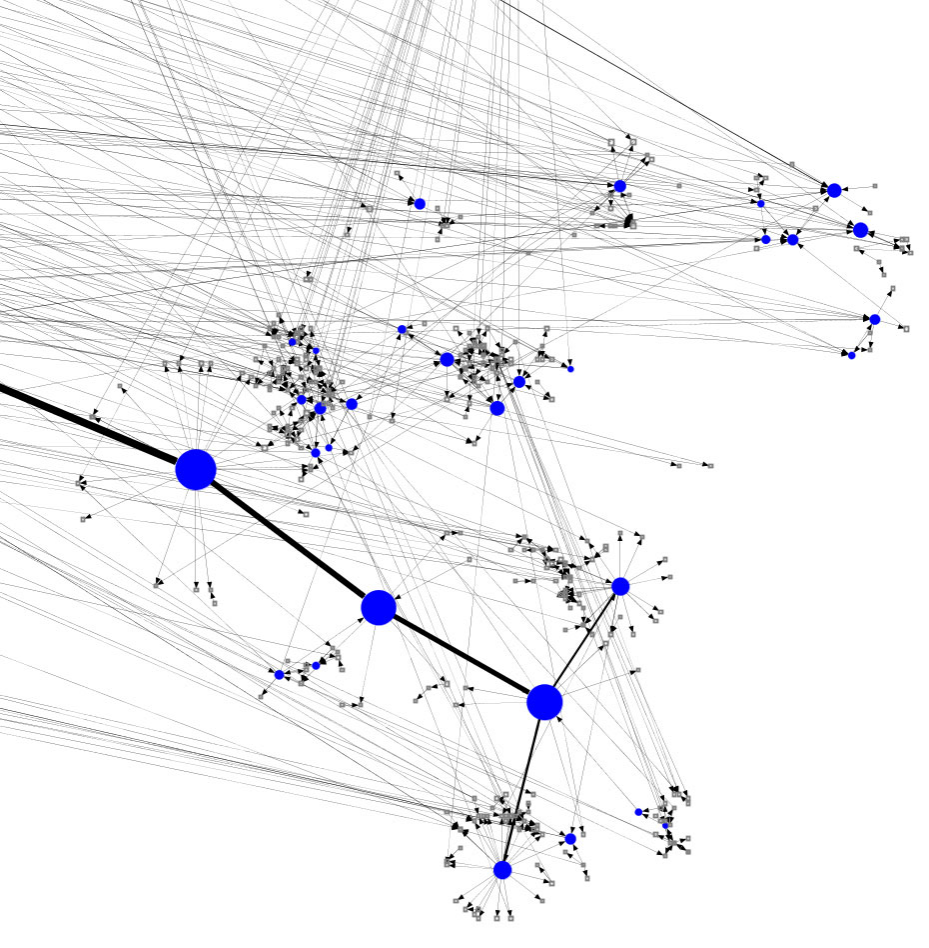
**Enlarged view of Betweenness layout algorithm**. A layout result of UO analysis network run on Cell Illustrator using betweenness.

We show layouts of UO Analysis using GEM-Tulip (Figure 3), Pajek (Figures 4 and 5), RSDFP-InterViewer (Figure 6) and our algorithm BFL on Cell Illustrator (Figure 7) [34,35]. The graph was not of extremely high degree; however, Pajek and InterViewer both produce layouts with no discernible network structure. Tulip with automatic sizing performs better, sorting all of the unconnected networks to the outside; however the program took eleven minutes, an order of magnitude more than any other program. Our betweenness-based algorithm was the fastest and also produced a readable layout. Our algorithm can naturally create a multi-scale layout, making low-betweenness nodes smaller to give space for large betweenness nodes and edges. Figure 8 shows an enlargement of a section of the graph, demonstrating this feature of our algorithm. Magnification shows that the complexity of the graph is simply stored at smaller sizes. In contrast, all other algorithms fail to create such a layering.

We also note that the runtime of this set was significantly lower (800 ms) for the betweenness algorithm compared to the others. The betweenness algorithm performs drastically better than others with sparse and multiscale datasets, while the competing algorithms have similar performance in randomly generated graphs.

### Betweenness is critical to BFL layout structure

**Figure 9:**
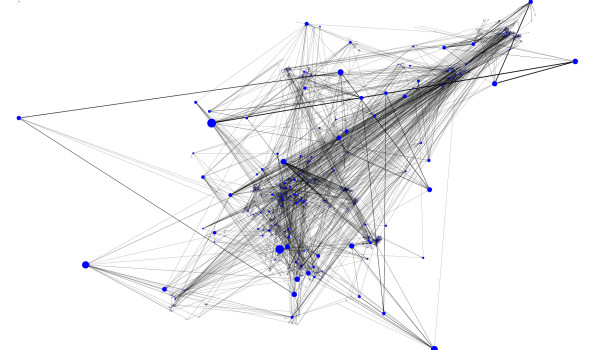
**Layout dependency on Betweenness**. Layout algorithm was run without betweenness modifications to the score. Scaling and sequential insertion were kept the same.

In order to show that betweenness enforces aesthetic constraints of density and compactness, we compared BFL to a modified version which did not weight scores based upon betweenness. Figures 7 and 9 respectively show the original and modified version of BFL. While the unweighted density, edge length and crossing parameters were similar in both runs, the unmodified BFL is visually superior because of its ability to force lower betweenness nodes to conform to larger betweenness nodes.

## Conclusion

Our BFL layout algorithm mainly achieved following two points: (i) the important elements (high betweenness nodes and edges) are emphasized in the resulting layout, (ii) the layout algorithm runs in real-time for large scale gene networks (around 10000 elements). For a graph with *n *nodes, this approach reduces the expected runtime of the algorithm to *O*(*n*^2^) when considering edge crossings, and to *O*(*n *log *n*) when considering only density and edge lengths. We also compared against fast graph layout algorithms and approaches requiring intensive optimizations. For gene networks, our algorithm was faster than all layout algorithms tested while providing readability on par with intensive optimization algorithms. We achieve a 1.4 second runtime for a graph with 4000 nodes and 12000 edges on a standard desktop computer. We will develop an effective tuning method for scaling parameters automatically in response to change in graph degree and optimize the algorithm further. We also intend to show that the layout algorithm provides a rough metric of functional relations, where betweenness separates functionally unrelated units and identifies hub genes.

## Authors' contributions

Basic idea was conceived by MN. Idea was developed and implemented by TH, with KK and MN advising. The manuscript was written by TH and revised by MN and KK. SM supervised throughout. Manuscript was read and approved by all authors.

## Supplementary Material

Additional file 1 **Network data**. UO analysis dataset used for layout comparison in GML, TUL, NET, and CSML file. CSML file has been stripped of all metadata not related to the graph structure. Zip file should be extracted for all data. Click here for file
